# Supplementary material for: Rapid turnover of life-cycle-related genes in the brown algae
Source: Genome Biol. 2019 Feb 14;20:35. doi: 10.1186/s13059-019-1630-6 (PMC6374913; doi:10.1186/s13059-019-1630-6)
Supplement: Supplementary file 3 — Protein alignments, corresponding Gblocks html file and CDS sequences for the lineage conserved gametophyte-biased genes. (ZIP 2109 kb) [file 13059_2019_1630_MOESM3_ESM.zip › Con_GA_alignments/Ec-20_002440.aa_ali.fasta-gb.htm]

Ec-20\_002440.aa.fasta.trans.aa\_ali.fasta


## Gblocks 0.91b Results

Processed file: **Ec-20\_002440.aa.fasta.trans.aa\_ali.fasta**  
Number of sequences: **2**  
Alignment assumed to be: **Protein**  
New number of positions: **303** (selected positions are underlined in blue)

```
                         10        20        30        40        50        60
                 =========+=========+=========+=========+=========+=========+
Ec-20_002440     MNASLLAALSSPVRGARTPVSN---ATAVSGKTKRNGP-KHTTGAGSTNNKENTTSSSSW
MSTRG.58733.1    MNASMLAALSSPG-GSKTPARRSGGAAFSTDKSKRAAAGAPGAGAGAGQNKENS---VS-
                 ############             ##########        ##########       


                         70        80        90       100       110       120
                 =========+=========+=========+=========+=========+=========+
Ec-20_002440     MAAAKTAAAVDGSRAPAFTALETPSTPGRMENLRVLIEEGKKAAAEGRLAAALRKYETAM
MSTRG.58733.1    --AAKPKANAGGSRAPAFTVPEAPSTPWRMENLRVLIEEGKKAAAEGRLSAALRKYETAM
                   ##########################################################


                        130       140       150       160       170       180
                 =========+=========+=========+=========+=========+=========+
Ec-20_002440     VTLPEAYKPRLRKKIARLEALLDDEEER--------------------------------
MSTRG.58733.1    VTLPEAYKPRLRNKIARLEARLDDEEARSPGTPARLSRKACREAAQSFGAKDPGPLDLLQ
                 ############################                                


                        190       200       210       220       230       240
                 =========+=========+=========+=========+=========+=========+
Ec-20_002440     ------------------------------------PRRSSPLARAFTGSRDAMPCVSG-
MSTRG.58733.1    VAVNDIAAAAAAAGGGGGGGSASGRLIAGGTFSSPSPRRSSPLVRGLAGAGDATPNASGV
                                                     ####################### 


                        250       260       270       280       290       300
                 =========+=========+=========+=========+=========+=========+
Ec-20_002440     TGRCSASPNDQDVGLDMAGLGDKMSASSLLA-TAASEPYRSPSR------------PRET
MSTRG.58733.1    AGKRAMTPRDQDLSFGIDRLGDRLTASSLTASTTAADPVGAPTSPVRSPLPASLQAARIN
                  ############################## ##########                  


                        310       320       330       340       350       360
                 =========+=========+=========+=========+=========+=========+
Ec-20_002440     -AGDHDHAR-GVSLSEEASPGA--G-EDGGYCGLGEAGPEEEEEEEQFDGESMDAQERLR
MSTRG.58733.1    RGDDCEQGQPGVFSAAEAGSAAGIGDEDDDHCGLGGAEEEAGSDEE---MEGLDTQERLR
                           ############    ####################    ##########


                        370       380       390       400       410       420
                 =========+=========+=========+=========+=========+=========+
Ec-20_002440     AMNRGLSGKLEKQVVDIMNQASSAELTDLHGIGPRRAQLILQTRAAAPFETLADLSRIGM
MSTRG.58733.1    EMNRGLSGKLEQQIVDIINKASSADLMELHGIGQQRAQLILKKRAEAPFETLADLKRINM
                 ############################################################


                        430       440       450
                 =========+=========+=========+========
Ec-20_002440     YPKQIEKMGRHNMLECLDVCSPVESSTREEAASAAAAL
MSTRG.58733.1    YEKEIWTLGRRNMLECLDVCR---STKPEDGAVVNNN-
                 ####################
```

```
Parameters used
Minimum Number Of Sequences For A Conserved Position: 2
Minimum Number Of Sequences For A Flanking Position: 2
Maximum Number Of Contiguous Nonconserved Positions: 8
Minimum Length Of A Block: 10
Allowed Gap Positions: None
Use Similarity Matrices: Yes
```

```
Flank positions of the 10 selected block(s)
Flanks: [1  12]  [26  35]  [44  53]  [63  148]  [217  239]  [242  271]  [273  282]  [311  322]  [327  346]  [351  440]  

New number of positions in Ec-20_002440.aa.fasta.trans.aa_ali.fasta-gb:  303  (66% of the original 458 positions)
```
